# Nomogram for spontaneous reduction in pediatric intussusception: a retrospective study

**DOI:** 10.3389/fped.2025.1571203

**Published:** 2025-07-09

**Authors:** Guangyu Wang, Zhongce Li, Guangqi Duan, Bangzhi Sui, Zhiyuan Jin, Zhenjie Chu, Rui Tang, Xiao Wang, Honglong Ma, Shiqin Qi, Jie Liu

**Affiliations:** ^1^ Department of Pediatric Surgery, Yijishan Hospital of Wannan Medical College, Wannan Medical College, Wuhu, China; ^2^ Department of General Surgery, Anhui Children's Hospital, Hefei, China

**Keywords:** intussusception, spontaneous reduction, nomogram, predictive model, children

## Abstract

**Objective:**

This study aimed to develop a nomogram to predict the probability of spontaneous reduction of intussusception (SROI) in pediatric patients.

**Methods:**

Clinical data of children diagnosed with intussusception and admitted to two hospitals in China from May 2023 to December 2024 were retrospectively analyzed. The eligible patients were randomly divided into the training and validation cohorts in a 7:3 ratio. The least absolute shrinkage and selection operator (LASSO) and multivariable logistic regression analyses were employed to identify essential variables for the development of the nomogram. The nomogram's performance was evaluated using receiver operating characteristic (ROC) curves, calibration curves, and decision curve analysis (DCA).

**Results:**

A total of 290 cases were included, of whom 114 patients underwent spontaneous reduction. The study identified six predictors of SROI: age, presence of bloody stool (bleed), comorbidities, mode of birth (MOB), intussusception region (right, left, and mid-abdomen), and length. The results demonstrate that older age and short-segment intussusception are significantly associated with an enhanced possibility of SROI. The nomogram demonstrated high discriminatory power with an area under the ROC curve (AUC) of 0.922 in the training cohort and 0.932 in the validation cohort. Calibration curves showed good agreement between predicted and observed outcomes. DCA indicated that the nomogram provided substantial net benefits for clinical application.

**Conclusion:**

The developed nomogram is a reliable and precise tool for predicting the likelihood of SROI in pediatric patients. It can assist clinicians in making treatment decisions, potentially reducing unnecessary invasive interventions and optimizing healthcare resource utilization.

## 1 Introduction

Intussusception refers to the invagination of a segment of the intestine into its own lumen, **including ileocolonic, ileocecal, and small intestinal intussusception.** This condition is one of the most common causes of acute abdomen in early childhood, especially in infants under 2 years old (1). If not promptly diagnosed and treated, it can lead to complications, such as intestinal obstruction and perforation, and even death (2, 3). Spontaneous reduction of intussusception (SROI) refers to the autonomous repositioning of invaginated bowel segments without clinical intervention, **which is more common in small intestinal intussusception** (4). This condition was first documented by Goldman et al. in 1940 (5). With the increasing reports of SROI in recent years (6–8), should therapeutic strategies be adjusted accordingly? In clinical practice, we have encountered multiple cases in which no intussusception was found during the enema procedure. In one case, no invagination was found during laparoscopic exploration for a patient with intussusception, and we ultimately attributed this to spontaneous reduction during anesthesia or the exploration process.

Therefore, we aimed to develop a predictive model to help physicians assess the probability of spontaneous reduction in patients, thereby guiding appropriate treatment decisions and safely avoiding interventions, optimizing resource use in select cases.

## 2 Patients and methods

### 2.1 Patients

**Figure 1:**
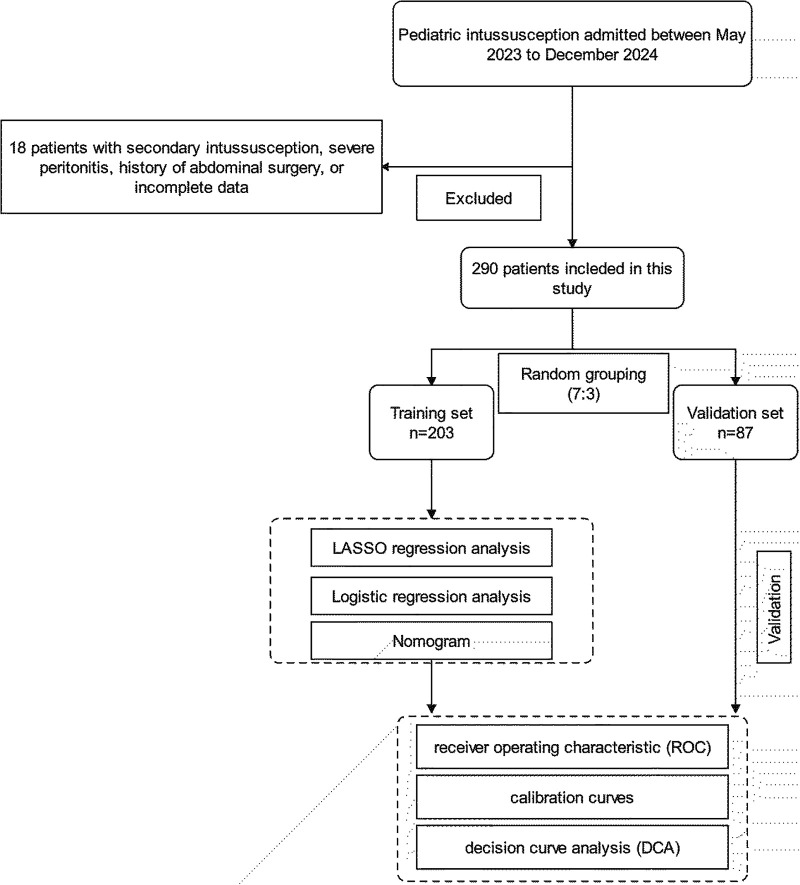
Flowchart of patient selection and nomogram construction.

This study was reviewed and approved by the ethics committee of Yijishan Hospital (Wuhu), Wannan Medical College, and the requirement for informed consent was waived in accordance with the Declaration of Helsinki. The clinical data of patients with intussusception who are admitted to the Yijishan Hospital and Anhui Children's Hospital, from May 2023 to December 2024, were obtained. The clinical diagnosis of intussusception was confirmed by ultrasound, showing a “target ring sign” or a “concentric circle sign” in the transverse section. The longitudinal section shows a “sleeve sign,” and intussusception length was measured by assessing the longitudinal distance from the entering to the returning limb. All patients underwent ultrasound diagnosis performed jointly by two experienced sonographers, **and agreement between the physicians was reached before a final diagnosis was made. Following initial assessment, conservative measures including hydration, manual repositioning, and close monitoring were implemented, with follow-up ultrasonography performed 1 h postintervention, repeated no more than twice. Patients who recovered spontaneously without invasive treatment (pneumatic air enema, hydrostatic enema, or surgical treatment) were recognized as having a self-resetting intussusception.** The inclusion criteria were as follows: (1) patients diagnosed with intussusception and (2) aged 0–14 years. The exclusion criteria were as follows: (1) patients with secondary intussusception due to underlying pathological lead points (e.g., tumors, polyps, or Meckel's diverticulum), (2) severe peritonitis signs requiring emergency surgery, (3) history of abdominal surgery, or (4) incomplete clinical data. These criteria were established to ensure a homogeneous study population and to minimize confounding factors that could influence the predictive accuracy of the logistic regression and nomogram models (Figure 1).

### 2.2 Data collection

The following clinical data were obtained: sex, age (month), weight (kilogram), symptoms (bloody stool, diarrhea, vomit), comorbidities (respiratory tract infection, rotavirus enteritis, or Henoch–Schönlein purpura), history of intussusception, mode of birth (natural birth, cesarean section), intussusception region (right, left, and mid-abdomen), mesenteric lymphadenopathy, DOS (hour), complete blood count (WBC, C-reactive protein, Neu%, Lymph%), and intussusception length (millimeter). The data on the outcome, i.e., SROI, were also collected.

### 2.3 Statistical analysis

The dataset collected from the Yijishan Hospital and Anhui Children's Hospital was randomly divided into training and validation cohorts at a 7:3 ratio, and the variables were compared. Non-normal data were presented as median (interquartile ranges). In the univariate analysis, the chi-square test or Fisher's exact test was used to analyze the categorical variables, while the Student's *t*-test or rank-sum test was used to examine the continuous variables. In the training cohort, the least absolute shrinkage and selection operator (LASSO) regression analysis was used to screen the potential predictors. These feature predictors selected in LASSO regression were further analyzed in a multivariate logistic regression to identify the significant predictors associated with SROI and build a nomogram (9). The performance of the model was assessed using the receiver operating characteristic (ROC) curve and calibration curve, with the area under the ROC curve (AUC) ranging from 0.5 (no discriminant) to 1 (complete discriminant). A decision curve analysis (DCA) was also performed to determine the net benefit threshold of prediction. To facilitate their incorporation into clinical practice, an interactive web-based dynamic nomogram application was built using Shiny. Results with a *p*-value of <0.05 were considered significant. All statistical analyses were performed using the R software (version 4.2.2), along with the MSTATA software (https://www.mstata.com).

## 3 Result

### 3.1 Patients' characteristics

In this study, a total of 308 patients with intussusception were initially selected. After excluding 18 patients with secondary intussusception, severe peritonitis, history of abdominal surgery, or incomplete data, a total of 290 eligible children were finally included in this study. Among the 290 cases, 182 were boys and 108 were girls, with a median age of 40 months. The entire cohort was randomly divided into a training set (*n* = 203) and a validation set (*n* = 87) at a 7:3 ratio. Among them, 114 patients experienced spontaneous reduction of intussusception, with 82 patients in the training set and 32 in the validation set. Statistical analysis showed no significant differences between the training and validation sets (*p* > 0.05). The baseline characteristics of the patients are shown in Tables 1 and 2.

**Table 1:** Baseline characteristics between the SROI and non-SROI groups.

Characteristic	SROI			*p*-value
	Overall, *N* = 290	No, *N* = 176	Yes, *N* = 114	
Age (month)	40 (24, 54)	34 (21, 49)	50 (39, 65)	<0.001
Weight (kg)	15.8 (12.0, 19.0)	15.0 (11.8, 17.5)	17.4 (15.5, 21.5)	<0.001
DOS (hour)	9 (5, 14)	10 (6, 20)	8 (5, 12)	0.007
WBC (x10^9^/L)	11.0 (8.5, 14.5)	10.8 (8.1, 14.2)	12.3 (9.4, 15.1)	0.033
CRP (mg/L)	3 (1, 10)	4 (2, 12)	3 (1, 8)	0.004
Neu (%)	79 (62, 87)	74 (56, 82)	84 (71, 92)	<0.001
Lymph (%)	16 (8, 30)	19 (11, 35)	13 (5, 22)	<0.001
Length (mm)	26 (21, 32)	29 (25, 37)	21 (17, 24)	<0.001
Sex				0.701
Girl	108 (37.2%)	64 (36.4%)	44 (38.6%)	
Boy	182 (62.8%)	112 (63.6%)	70 (61.4%)	
Bleed				<0.001
No	248 (85.5%)	136 (77.3%)	112 (98.2%)	
Yes	42 (14.5%)	40 (22.7%)	2 (1.8%)	
Diarrhea				0.592
No	272 (93.8%)	164 (93.2%)	108 (94.7%)	
Yes	18 (6.2%)	12 (6.8%)	6 (5.3%)	
Vomit				0.714
No	48 (16.6%)	28 (15.9%)	20 (17.5%)	
Yes	242 (83.4%)	148 (84.1%)	94 (82.5%)	
Comorbidities				0.076
No	246 (84.8%)	144 (81.8%)	102 (89.5%)	
Yes	44 (15.2%)	32 (18.2%)	12 (10.5%)	
HOI				0.045
No	244 (84.1%)	142 (80.7%)	102 (89.5%)	
Yes	46 (15.9%)	34 (19.3%)	12 (10.5%)	
MOB				<0.001
CS	170 (58.6%)	120 (68.2%)	50 (43.9%)	
NB	120 (41.4%)	56 (31.8%)	64 (56.1%)	
region				<0.001
Left	56 (19.3%)	16 (9.1%)	40 (35.1%)	
Mid	32 (11.0%)	12 (6.8%)	20 (17.5%)	
Right	202 (69.7%)	148 (84.1%)	54 (47.4%)	
MLA				0.841
No	114 (39.3%)	70 (39.8%)	44 (38.6%)	
Yes	176 (60.7%)	106 (60.2%)	70 (61.4%)	

HOI, history of intussusception; MOB, mode of birth; MLA, mesenteric lymphadenopathy; DOS, duration of symptoms; SROI, spontaneous reduction of intussusception; NB, natural birth; CS, cesarean section; CRP, C-reactive protein; WBC, white blood cell count.

**Table 2:** Baseline characteristics of patients in the training and validation cohorts.

Characteristics	Training cohort			Validation cohort		
	No, *N* = 121	Yes, *N* = 82	*p*-value	No, *N* = 55	Yes, *N* = 32	*p*-value
Sex			0.881			0.358
Girl	47 (39%)	31 (38%)		17 (31%)	13 (41%)	
Boy	74 (61%)	51 (62%)		38 (69%)	19 (59%)	
Bleed			<0.001			0.012
No	94 (78%)	81 (99%)		42 (76%)	31 (97%)	
Yes	27 (22%)	1 (1%)		13 (24%)	1 (3%)	
Diarrhea			0.350			0.623
No	111 (92%)	78 (95%)		53 (96%)	30 (94%)	
Yes	10 (8%)	4 (5%)		2 (4%)	2 (6%)	
Vomit			0.179			0.285
No	14 (12%)	15 (18%)		14 (25%)	5 (16%)	
Yes	107 (88%)	67 (82%)		41 (75%)	27 (84%)	
Comorbidities			0.039			0.928
No	98 (81%)	75 (91%)		46 (84%)	27 (84%)	
Yes	23 (19%)	7 (9%)		9 (16%)	5 (16%)	
HOI			0.129			0.207
No	100 (83%)	74 (90%)		42 (76%)	28 (88%)	
Yes	21 (17%)	8 (10%)		13 (24%)	4 (13%)	
MOB			0.001			0.012
CS	81 (67%)	36 (44%)		39 (71%)	14 (44%)	
NB	40 (33%)	46 (56%)		16 (29%)	18 (56%)	
Region			<0.001			<0.001
Left	12 (10%)	29 (35%)		4 (7%)	11 (34%)	
Mid	9 (7%)	15 (18%)		3 (5%)	5 (16%)	
Right	100 (83%)	38 (46%)		48 (87%)	16 (50%)	
Age (month)			<0.001			<0.001
Mean ± SD	35 ± 20	50 ± 22		36 ± 22	58 ± 31	
MLA			0.498			0.496
No	50 (41%)	30 (37%)		20 (36%)	14 (44%)	
Yes	71 (59%)	52 (63%)		35 (64%)	18 (56%)	
Weight (kg)			<0.001			0.007
Mean ± SD	14.9 ± 5.1	18.2 ± 5.3		15.6 ± 5.4	19.7 ± 7.2	
DOS (hour)			0.009			0.382
Mean ± SD	14 ± 11	10 ± 9		13 ± 11	11 ± 11	
WBC (x10^9^/L)			0.055			0.213
Mean ± SD	11.4 ± 4.4	12.7 ± 4.9		11.4 ± 4.6	13.0 ± 6.1	
CRP (mg/L)			0.004			0.059
Mean ± SD	10 ± 17	5 ± 6		9 ± 15	5 ± 8	
Neu (%)			<0.001			0.006
Mean ± SD	69 ± 19	79 ± 15		68 ± 18	79 ± 16	
Lymph (%)			<0.001			0.021
Mean ± SD	24 ± 17	16 ± 14		24 ± 15	16 ± 14	
Length (mm)			<0.001			<0.001
Mean ± SD	31 ± 9	21 ± 4		33 ± 11	21 ± 5	

HOI, history of intussusception; MOB, mode of birth; MLA, mesenteric lymphadenopathy; DOS, duration of symptoms; SROI, spontaneous reduction of intussusception; NB, natural birth; CS, cesarean section; CRP, C-reactive protein; WBC, white blood cell count.

### 3.2 Predictive model

**Figure 2:**
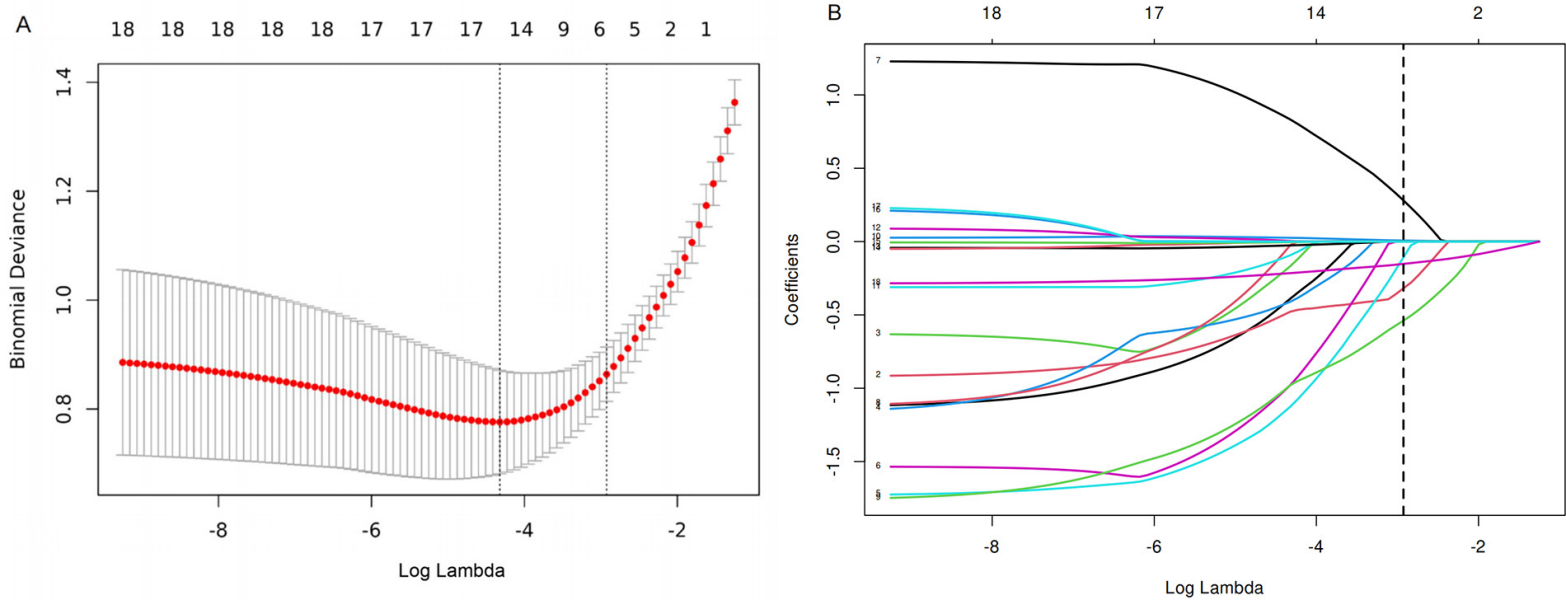
**(A)** The optimal parameter (*λ*) selection in the LASSO model employed 10-fold cross-validation using a minimum criteria approach. Among these values, *λ* = 0.053, corresponding to a logarithm of *λ* = −2.926, was selected as the optimal choice. **(B)** LASSO coefficient profiles of clinical features. The plot was created using a logarithmic scale for the *λ* values. This optimal *λ* value led to the identification of six features with nonzero coefficients.

LASSO regression was used for preliminary screening in the training cohort to identify potential predictors, avoid overfitting, and enhance the robustness of the model. The most regularized and parsimonious model, with a 10-fold cross-validated error within one standard error of the minimum, included six variables, i.e., age, MOB, bleed, intussusception region, comorbidities, and length (Figures 2A,B). The coefficients of LASSO regression analysis are shown in Table 3.

**Table 3:** The coefficients of LASSO regression analysis.

variable	Coefficient
(Intercept)	3.499550173
Sex_boy	0.000000000
Age	0.007022513
Weight	0.000000000
DOS	0.000000000
Bleed_yes	−0.318517754
Diarrhea_yes	0.000000000
Vomit_yes	0.000000000
Comorbidities_yes	−0.107995891
HOI_yes	0.000000000
MOB_NB	0.280737928
WBC	0.000000000
CRP	0.000000000
Neu	0.000000000
Lymph	0.000000000
Region_mid	0.000000000
Region_right	−0.539115196
MLA_yes	0.000000000
Length	−0.152156537

HOI, history of intussusception; MOB, mode of birth; MLA, mesenteric lymphadenopathy; DOS, duration of symptoms; SROI, spontaneous reduction of intussusception; NB, natural birth; CS, cesarean section; CRP, C-reactive protein; WBC, white blood cell count.

**Figure 3:**
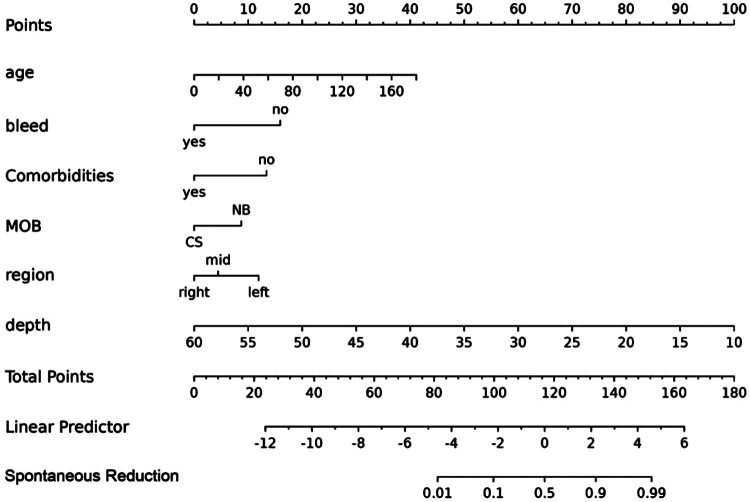
The nomogram for predicting the probability of SROI. Different levels of each variable correspond to the points. Finally, the total points and probability of SROI can be calculated.

These feature predictors were subsequently included in the multivariate logistic regression. As expected, bloody stools (OR = 0.13, 95% CI: 0.01–1.13), comorbidities (OR = 0.18, 95% CI: 0.04–0.68), region [right abdomen (OR = 0.21, 95% CI: 0.07–0.62)], and increased length (OR = 0.77, 95% CI: 0.70–0.84) were associated with a lower likelihood of SROI. Older age (OR = 1.03, 95% CI: 1.03–1.06) and natural birth (OR = 3.08, 95% CI: 1.25–8.04) were associated with an increased probability of SROI (Table 4). The final logistic model included six independent predictors (age, bleed, comorbidities, MOB, region, and length) and was developed as a simple-to-use nomogram (Figure 3) (available at https://wangguangyu.shinyapps.io/dynnomapp/).

**Table 4:** Results of multivariate logistic regression for the training cohort.

Characteristic	*N*	Event *N*	OR	95% CI	*p*-value
Age	203	82	1.03	1.01, 1.06	0.014
Bleed					
No	175	81	—	—	
Yes	28	1	0.13	0.01, 1.13	0.113
Comorbidities					
No	173	75	—	—	
Yes	30	7	0.18	0.04, 0.68	0.014
MOB					
CS	117	36	—	—	
NB	86	46	3.08	1.25, 8.04	0.017
Region					
Left	41	29	—	—	
Mid	24	15	0.38	0.08, 1.80	0.227
Right	138	38	0.21	0.07, 0.62	0.007
Length	203	82	0.77	0.70, 0.84	<0.001

OR, odds ratio; CI, confidence interval; MOB, mode of birth.

**Figure 4:**
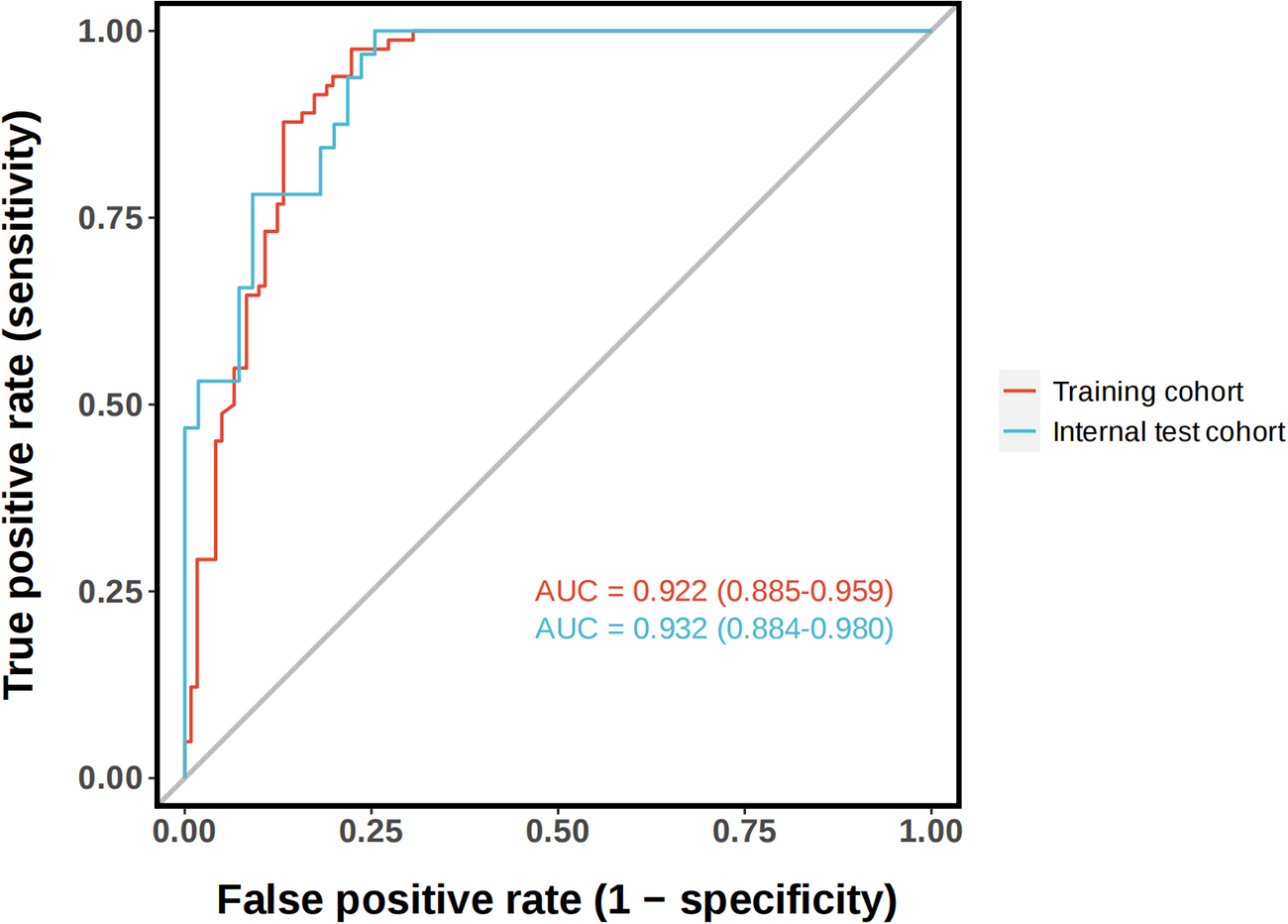
The receiver operating characteristic (ROC) curves and area under the ROC curve (AUC) of the nomogram prediction model. The training cohort, AUC = 0.922 (95% CI: 0.885−0.959). The validation cohort, AUC = 0.932 (95% CI: 0.884−0.980).

The ROC curve analysis in this study showed that the model was effective in predicting the probability of spontaneous reduction of intussusception in children. The area under the curve (AUC) for the training cohort was 0.922 (95% CI: 0.885–0.959) and 0.932 (95% CI: 0.884–0.980) for the validation cohort (Figure 4).

**Figure 5:**
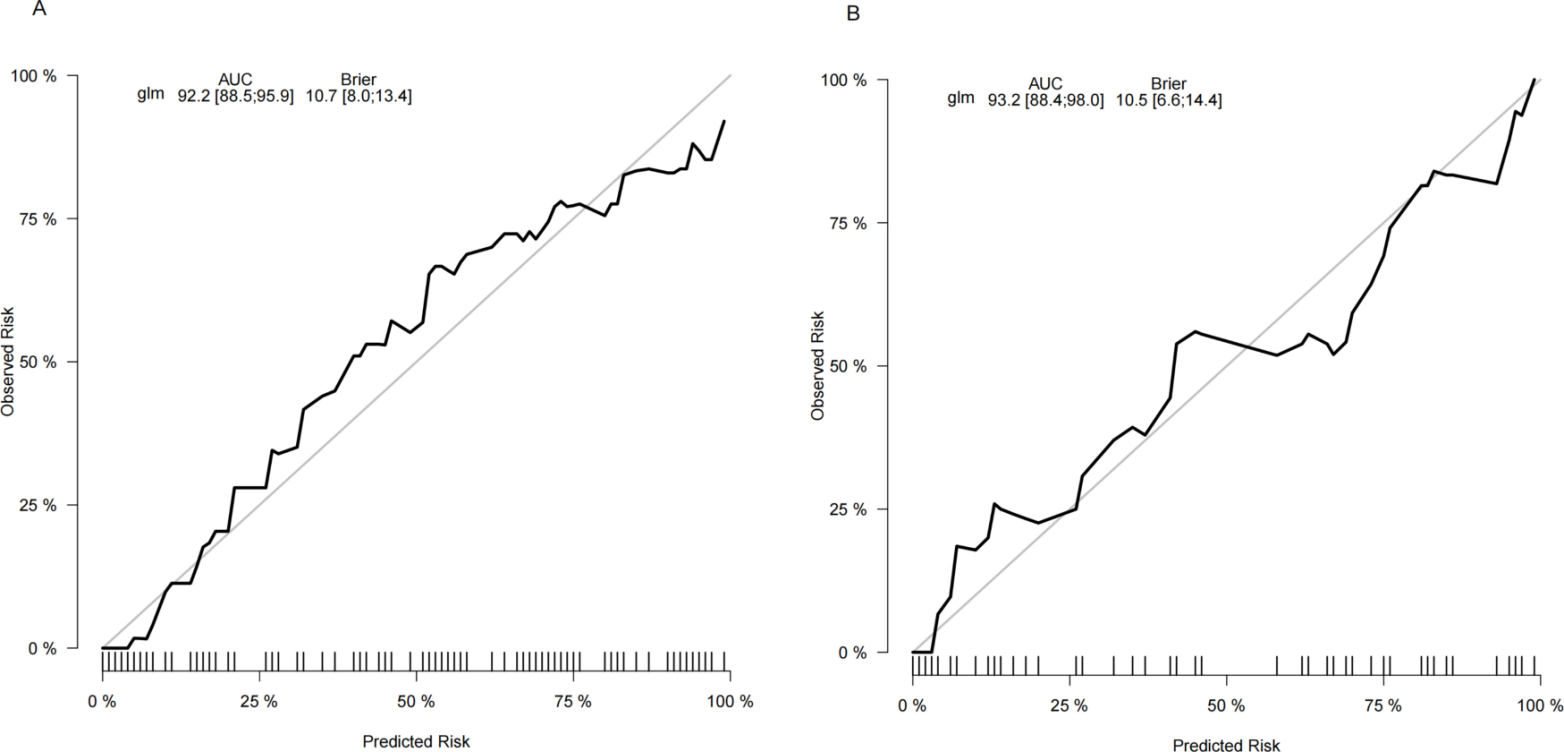
Calibration curve of the nomogram. The *y*-axis represents the actual SROI rate. The *x*-axis represents the predicted risk of SROI. **(A)** The training cohort. **(B)** The validation cohort.

The calibration plots of the nomogram in different cohorts are plotted in Figures 5A,B, which demonstrate a good correlation between the observed and predicted SROI. The results showed that the nomogram was still valid for use in the validation sets, and the calibration curve of this model was relatively close to the ideal curve, which indicates that the predicted results were consistent with the actual findings.

### 3.3 Clinical use

**Figure 6:**
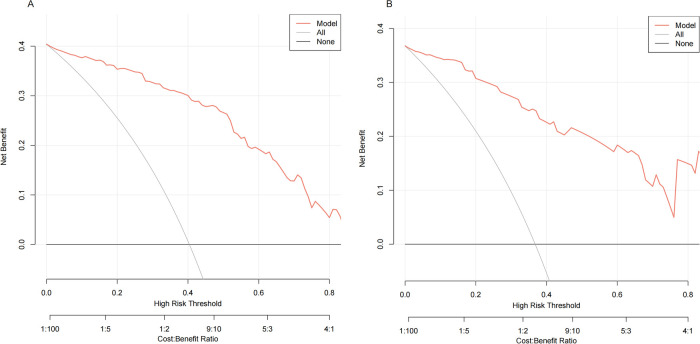
Decision curve analysis (DCA) of the nomogram. The black line represents the assumption of no patient having SROI, while the gray line assumes that all patients experienced SROI. The red line corresponds to the nomogram. The DCA demonstrated that the nomogram could provide a higher overall net benefit. **(A)** The training cohort. **(B)** The validation cohort.

**Figure 7:**
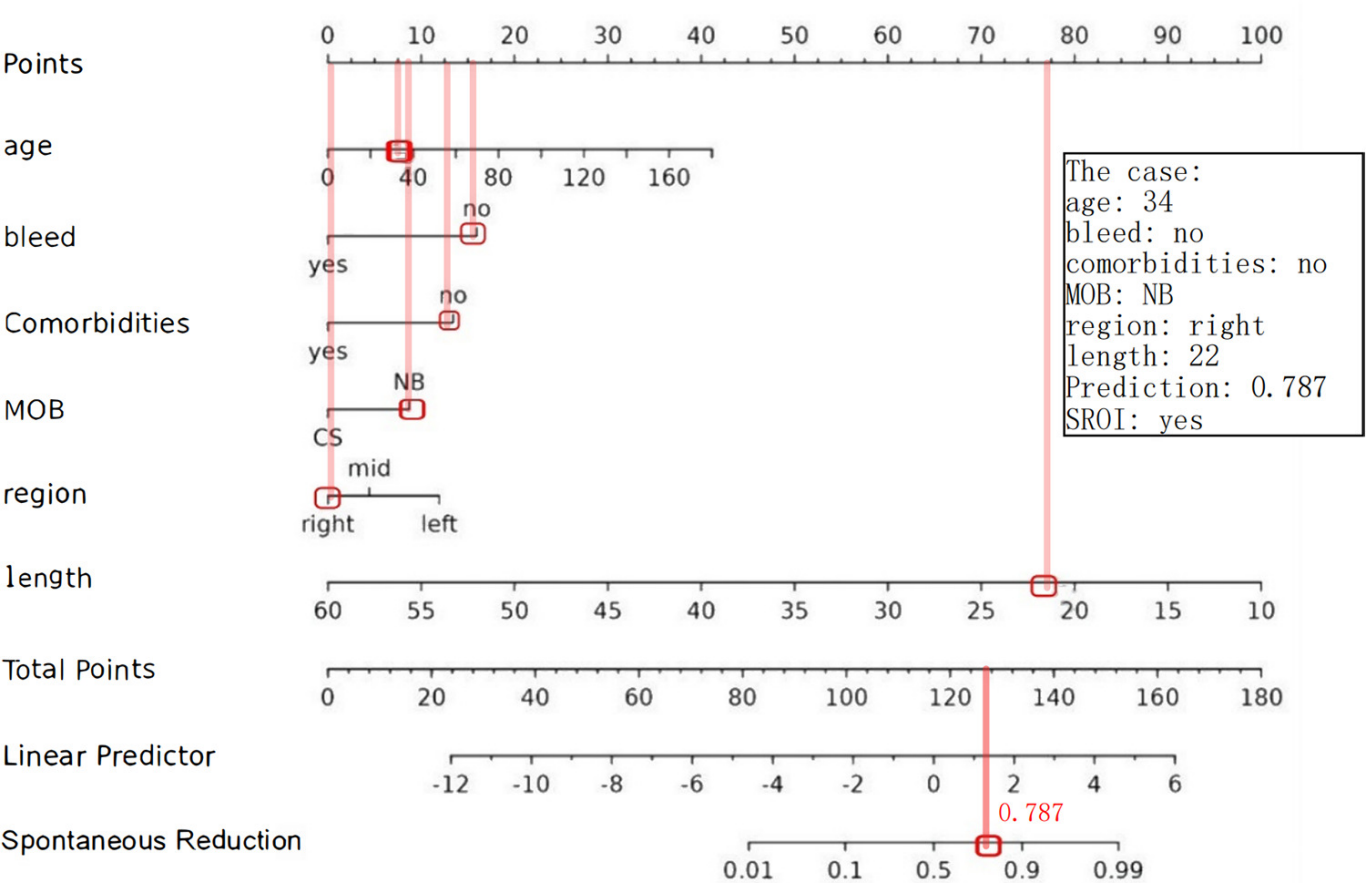
Example of adopting a nomogram. The estimated probability of SROI for this case was 0.787, and this child eventually had an SROI.

The following figure displays the DCA curves related to the nomogram (Figures 6A,B). The DCA curve demonstrates that the nomogram provides significant clinical utility across a wide range of threshold probabilities, particularly between 10% and 50% probability thresholds. In clinical practice, by setting the threshold probability at 50%, patients with a predicted probability higher than this threshold may be managed with a conservative approach (see example in Figure 7).

## 4 Discussion

Intussusception, characterized by the invagination of a bowel segment into its own lumen, is the second most common cause of acute abdomen in children, following acute appendicitis (10). Spontaneous reduction of intussusception refers to the unassisted reversion of the intestine to its normal anatomical position without any medical intervention. The nomogram is a predictive model extensively utilized in clinical research, integrating key prognostic factors to construct a graphical calculator that visualizes the probability of clinical outcomes (11). In this study, we developed a nomogram to predict the likelihood of spontaneous reduction of intussusception (SROI) in pediatric patients and comprehensively evaluated its performance. Retrospective analysis of data from 290 children identified six independent predictors significantly associated with SROI: age, presence of bloody stool, comorbidities, mode of birth, region, and length of intussusception. The model demonstrated outstanding discriminatory power, with area under the curve (AUC) values of 0.922 and 0.932 in the training and validation cohorts, respectively. Furthermore, calibration curves and decision curve analysis (DCA) confirmed the model's excellent calibration accuracy and robust clinical utility. These findings suggest that the nomogram serves as a reliable and precise tool for clinicians to evaluate the probability of SROI, facilitating more informed treatment decisions. By minimizing unnecessary invasive interventions, the tool reduces patient discomfort and optimizes healthcare resource utilization.

Previous research has concentrated on predicting the outcomes of pneumatic reduction. Kim et al.'s (12) meta-analysis of predictors of enema failure in children with intussusception also identified factors such as bloody stools, age under 1 year, increased length, and infections that are commonly associated with a greater chance of treatment failure. Guogang et al. (13) considered infections and increased length as risk factors for hydrostatic reduction failure in intussusception. Our results also show that these factors are tied to a lower likelihood of spontaneous reduction.

The present study showed a higher rate of spontaneous reduction in older children, which may be closely related to the developmental maturity of the enteric nervous system. Studies have shown that the intestinal intermuscular plexus (Auerbach's plexus) in infants and young children is gradually perfected during the first 2–3 years of life, and its ability to regulate rhythmic contraction and relaxation of the intestine increases with age (14). In addition, the mesenteric fat pad is thicker in older children, which may reduce mechanical strangulation of the invaginated bowel segments through a cushioning effect (15).

Previous studies have shown that intussusception is strongly associated with certain viral infections, especially rotavirus and adenovirus (16, 17). In the present study, there were 44 patients with comorbidities (respiratory tract infection, rotavirus enteritis, or anaphylactic purpura), and children with these comorbidities were less likely to undergo spontaneous reduction (OR = 0.18, 95% CI: 0.04–0.68, *p* = 0.014), suggesting that concomitant illnesses may have a detrimental effect on spontaneous reduction. These concomitant diseases may affect the incidence of intussusception and the likelihood of reorientation by inducing a systemic inflammatory response (e.g., elevated IL-6 and TNF-α) that activates intestinal mast cells, releases histamine and proteases, leads to smooth muscle spasm and lymphatic reflux obstruction, and either increases intestinal wall tension or leads to intestinal dysfunction (18, 19). However, due to the limited sample size, there are constraints in grouping multiple diseases together. To address this, we are continuing to expand the sample size and plan to conduct more rigorous studies using multicenter, large-sample data in the future.

There are a few previous studies on the relationship between mode of birth and SROI. In the present study, we found a positive correlation between vaginal delivery and SROI (OR = 3.08, 95% CI: 1.25–8.04, *p* = 0.017), which may be attributed to the differences in early colonization of the gut flora, as newborns delivered vaginally can rapidly establish a probiotic-dominated intestinal microecology by being exposed to the maternal maternally delivered flora (e.g., *Lactobacillus* and *Bifidobacterium*) (20). These florae enhance intestinal epithelial barrier function by metabolizing short-chain fatty acids (e.g., butyric acid) and modulate the frequency of intestinal smooth muscle contractions (21). In contrast, cesarean-section babies have a reduced diversity of intestinal flora due to the lack of this process, which may lead to immune imbalances and peristaltic disturbances, increasing the risk of persistent strangulation after intussusception (22). Established studies have focused more on the impact of birth mode on other pediatric disorders, but its role in intussusception has not been extensively studied. The results of this study suggest that birth mode may be an important clinical predictor. This is an intriguing finding, but the link to gut flora is speculative at this stage. We will conduct a comprehensive and in-depth follow-up study on this discovery.

Previous studies have shown that the site of intussusception significantly affects the probability of its restoration (23). The low repositioning rate of right-sided intussusception (OR = 0.21, 95% CI: 0.07–0.62, *p* = 0.007) was associated with their anatomical characteristics. Right-sided intussusception tends to be ileocecal and ileocecocolic, often involving the ileocecal valve, which is rich in lymphatic tissue and has a narrow lumen, which tends to form a “flap effect,” and prevents the passage of the contents and accelerates ischemia (24). In addition, branches of the ileocolic artery are the terminal blood supply, making the region more susceptible to irreversible damage. **SROI is more common in small intestinal intussusception. Our study included 124 cases (42.7%) of small bowel intussusception, of which 82 (66.1%) exhibited SROI. Given the significant influence of intussusception type on SROI, we will expand our sample size for future research focusing on different intussusception types.**

Length was a strong correlate of SROI, with a significantly higher probability of SROI for each 1 mm decrease in length (OR = 0.77, 95% CI: 0.70–0.84, *p* < 0.001). Anatomical studies have shown that shallow intussusception usually involve only the mucosa and submucosa, whereas deep intussusception tends to compress mesenteric vessels, leading to ischemic injury and resistance to repositioning, and that the length is positively correlated with the absence of blood flow signals to the intestinal wall, with the latter being an important predictor of repositioning failure (25, 26). **The maximum diameter is also an important factor. However, since length and diameter are strongly correlated and collinear, as the invagination deepens, more tissue is involved, increasing the maximum diameter. So, we included only one to avoid redundancy and enhance model stability.**

The prediction model has important implications for the clinical management of intussusception in children. Accurate prediction of SROI can help physicians optimize treatment strategies. For example, invasive operations (e.g., enema repositioning or surgery) can be appropriately delayed in children with a high probability of SROI to reduce unnecessary medical interventions and their associated risks.

This study still has some limitations. This was a retrospective study with a small sample size and a lack of an external validation set. **As per the empirical 10-events-per-parameter (EPP) rule, our study achieved the minimum sample size of 180, while Riley's method suggests a minimum of 330 samples** (27). Although LASSO regression was used to reduce the risk of variable overfitting, the small sample size (*n* = 290), especially the small number of positive cases for SROI (*n* = 114), may affect the stability and accuracy of the model. Future studies should enhance the robustness of the model by expanding the sample size and validating with multicenter data. In addition, this study failed to incorporate some possible potential confounders, such as patients' nutritional status and immune level, which may have an impact on the incidence of SROI.

## 5 Conclusion

In this study, we developed and validated a nomogram for predicting the spontaneous resolution of intestinal intussusception in children. The model incorporates risk factors such as age, blood stool, mode of birth, comorbidities, and length and site of intussusception and has been validated in-house as a useful tool for assessment. The predictive model developed has some clinical applications.

## Data Availability

The raw data supporting the conclusions of this article will be made available by the authors, without undue reservation.
